# Report of Two Cases

**Published:** 1866-09

**Authors:** A. J. Shaffer

**Affiliations:** Lawrenceville, Ga.


### ARTICLE III.

*Report of Two Cases.* By A. J. SHAFFER, M. D., of Lawrenceville, Ga.

CASE 1.—Mr. E. C., aged 47 years, hight five feet ten inches, in good health, and would weigh one hundred and sixty pounds; of sanguine temperament, fair hair, and small blue eyes; had served three years in the Confederate army.

March 20th.—Was working on the farm, hauling rails and making fence. His dinner, which consisted of cornbread, bacon, and boiled peas, was sent to him, of which he ate moderately. After resting a few minutes, he resumed his work, making fence, and in the act of lifting a heavy oak rail upon a high fence, was suddenly taken with a very severe and excruciating pain in the symphysis pubis, (or, as he expressed it, in the middle of the cross bones,) with a pressing and persistent desire to evacuate the contents of the bowels, but could pass nothing. He vomited quite freely sundry times, and was conveyed home, when his physician, Dr. B. F. Freeman, was sent for. Supposing it to be cramp colic, caused by eating improper diet, the doctor addressed his treatment accordingly. He gave active cathartics, combined with opium; warm fomentations to the bowels, &c., &c. The cathartics were frequently repeated, and operated freely in the course of twenty-four hours, which caused the most excruciating pain. So severe were the pains caused by the act of defecation, that it required the assistance of three or four strong men to hold him. The matter discharged was of a healthy character and natural in appearance, only as modified by the influence of medicine, except the first operation, which was accompanied by a small portion of blood, perhaps ten drops. There was no difficulty at any time in passing water, which was of a healthy appearance. This is a short history of the case, from the time he was taken, up to the time that I saw him, April 4th, when the following pathological phenomena presented: Transient pains in the small intestines, most severe in the ilium and symphysis pubis; the abdomen very much swollen in its whole extent, from the os pubis to the ensiform cartilage. The parieties were unyielding to moderate pressure, yet heavy manipulation produced no pain in any part of the abdomen. On application of the ear, gaseous sounds could be heard, passing with great velocity through the intestinal canal. No gas was discharged. Percussion over or in the track of the small intestines would elicit sounds similar to that produced by pouring water out of a six ounce vial, but partaking a little more of the metallic click. These sounds were louder and heavier over the lower part of the ilium and sacrum. The sounds in the last named locality would strongly impress one that they were caused by water agitated in the peritoneal cavity. His breathing was quite imperfect, in consequence of the swelling of the abdomen. On percussion, there was very little resonance in any part of the chest except over the apex of each lung. The heart labored hard; the pulse eighty-five and small in the radial artery; the countenance anxious; the tongue rather red and moist; the posterior part having yellow coat; very little thirst; micturation free; urine nearly natural in appearance.

My first examination was for hernia; but failing to detect any symptom of that derangement by external manifestations, I injected about three pints of salt water into the bowel, which was attended with a good deal of pain, as the water was forced into the bowel. It was discharged in about two minutes, and voided with a considerable force, accompanied by gas. The discharge of enema was followed by about a half drachm of thin mucus. The all-important point now was to determine, from anatomical relations and pathological symptoms, which of the many obscure, accidental abdominal diseases, to which the intestinal tube is at all times exposed, was present. In considering the attitude of the patient at the time of the attack, and the great muscular effort called for, not only of the upper extremities, but also of the abdominal muscles, in the act of placing a heavy green oak rail upon a high fence, and the symptoms of disease following the act, is it not reasonable to suppose that it gave rise to one of the numerous and obscure herniæ to which the intestines are liable, which had become partially strangulated, and might have become permanently so, had it not been for the free exhibition of active purgative medicine, causing active paristaltic motion of the bowels?

Mr. Erichsen, in his valuable work on Surgery, says, that "active or acute intestinal obstruction may arise in various ways. When of a mechanical origin, it most commonly occurs in consequence of the formation of an internal hernia, which becomes suddenly strangulated—a portion of gut slipping through an aperture in the mesentery, or omentum, or becoming constricted by bands, adhesions, or diverticula, stretching across from one side of the abdomen to the other. In other cases again it may occur from invagination, the upper portion of the intestine slipping into the lower, or by a portion of gut becoming twisted upon itself and thus forming a volvulus, owing to the mesentery, or mesocolon, being unusually long, and allowing a half twist to take place, in consequence of which complete obstruction occurs." Of the symptoms, he says: "Acute intestinal obstructions, when arising from a mechanical cause, such as the formation of an internal hernia, or volvulus, are always characterized by very marked vital depression. Constipation is present from the very first, but this symptom is not the most prominent one, and those that result are evidently, as in an ordinary case of strangulated hernia, the consequence of the injury inflicted upon the intestine, rather than of the mere mechanical obstacle to the onward passage of the feces. At the moment of the occurrence of the attack, the patient is usually seized with a sudden feeling of something wrong having taken place in the abdomen; or he is struck with intense *pain at one point.* There may be sudden syncope, though most usually the depression of vital power does not amount to this. Vomiting speedily occurs—at first, of the contents of the stomach, but after a time of a stercoraceous character. The abdomen becomes swollen and tender; the intestines being blown up with flatus, so as to give rise to immense tympanitic distension rolling over one another and occasioning a loud rumbling and gurgling noise."

The general symptoms above enumerated, characterizing intra-abdominal hernia, are most strongly set forth in the patient before us, which, I have no doubt, was omental hernia, as I think the sequel of the case will prove. But the point in the case, which I wish more particularly to call attention to, is the very severe and persistent pain in the symphysis pubis, which was the first monitor to warn the patient that there was something wrong in the abdomen, and it was several days before pain was felt elsewhere; after which time he would at times have transitory pain in the lower part of the abdomen. Another prominent diagnostic symptom was the excruciating pain produced in the os pubis, when the cathartics first operated. There was evidently no disease in that bone, to be felt or seen. I can account for it only upon one physiological principle, that of reflex action. Drs. B. F. Freeman and T. K. Mitchell, after having thoroughly examined the case, gave it as their opinion, that the disease was inflammation of the bowels, predicating their diagnosis upon the above enumerated symptoms; in which decision I acquiesced under protest. The treatment was proposed according to that opinion. A large blister to the hypogastrium, balsamic emulsion, blue pills, light diet, &c., &c., were prescribed, and the case left in the care of Dr. F.

April 15th.—I was resummoned in consultation with Dr. F. I found the patient in a more prostrated condition—the symptoms all more aggravated. The swelling of the abdomen had greatly increased. The gaseous sounds could be heard spouting up and down the bowels without difficulty. By raising the chest, a gurgling noise could be heard descending, until it would all settle in the lower part of the bowels; then by heavy percussion over the ilium, a loud gurgling click, similar to that made by air and water, could be heard at a distance of ten yards. He was suffering a good deal from partial aphonia, in consequence of the enlargement of the abdomen and the reduced caliber of the chest's cavity—the bowels rather constipated—opiates had been freely given to relieve pain, which persisted in the old locality—micturates freely, and the water healthy in appearance. I was sent for to tap the patient for dropsy, and after a careful examination, I was confident that there was water in the peritoneal cavity, as the symptoms indicating that condition were present, even that of the wave. By the use of a crownpointed lancet, an opening was made into the peritoneal cavity, through the linea alba. I was no little surprised, after piercing the peritoneum, that no water escaped by the side of the lancet; and I supposed that it would flow when the catheter (female) was inserted; but after having used it to its full length and gently probing every part of the cavity, there was not more than ten or a dozen drops of fluid escaped, and on withdrawing it, was found filled with fluid, thick and of a strong alkaline taste and reaction. It is not necessary to state that this dry tapping was a little mortifying to our feelings, as a large number of persons felt disappointed in failing to witness a stream of water flowing from the abdomen. I know that some of the profession would say, that this tapping was quite unnecessary—that the existence or non-existence of water in the peritoneal cavity is so easily diagnosed that there is no excuse for mistake. In uncomplicated cases, that is true; but I will leave the symptoms in this case to plead my apology.

But was there not most valuable negative information gained by this little operation? I think it quite as important in all cases, simple as well as complicated, to consider well all the negative symptoms of a disease, as in most of cases it is only by debating the negative symptoms that we can intelligently arrive at a correct affirmative evidence of diseases. Hence the negative information, furnished in this case by a simple operation, proved to be most valuable. All surgeons are acquainted with the fact, that in strangulated hernia, large quantities of water will accumulate in a remarkably short space of time. I cannot say positively that this case was hernia; but it was evidently a mechanical difficulty of the bowels, and I am forced to this conclusion from the first symptoms of the disease and the sequel in convalescence. The following treatment was now adopted:

Hydr. chlo. mit. ℈iiss.

Jalap, - - - ℈iiss.

Mix and divide into twelve powders—one to be taken every two hours. An active dose of sulph. magnesia to be taken four hours after taking the last powder.

Tinct. of digitalis, in the dose of ten drops, was given every six hours, to be increased one drop each day. Opium to be given occasionally, to relieve pain and to prevent the powders from operating too actively. Blister to the epigastrium; light nutritious diet, etc.

April 19th.—Has taken the medicine according to direction, except one of the powders. After taking the ninth portion, he became severely nauseated and threw up large quantities of dark bile, and was actively purged at the same time. The discharges per rectum were copious, of dark, bilious matter. The blister drew well and discharged a great deal of water. The swelling of the abdomen is very much reduced, the parieties presenting a wrinkled appearance, and soft to the touch; breathes free and easy, as the lungs are freed from the encroachment of the diaphragm upon the thoracic cavity; pulse sixty per minute and of good size; tongue moist and natural in appearance; has very little pain; has passed more water than usual; no symptoms of ptyalism; he thinks that he will get well. Prescribed the following:

Hydr. chlo. mit. gr.xxiv.
Jalap, - - - gr.xxiv.

Mix and divide into six powders—one to be taken night and morning, followed by an active dose of sulph. magnesia. Tinct. of digitalis, three times per day in the dose of ten drops.

April 27th.—Patient sat up most of the day.

June 25th.—Convalescence complete.

Case 2.—Mrs. E. D., of this county, aged forty-five, of light form and in good health, would weigh one hundred and twenty-five pounds; unmarried, and had one child at twenty-five; ceased menstruating at forty-two.

In January, 1863, became anasarcous.

April 10th.—I tapped her and drew from the abdomen two and a half gallons of water. By the use of a No. 4 needle, I drew from the legs one gallon and a half. Since April 10th, 1863, up to this time, August 1st, 1866, she has been tapped seventy-two times, and had drawn from the abdomen two hundred and twenty gallons of water.

I have been using the needle for nine years in all dropsical cases of the legs. In one case, by the use of a No. 3 needle, I drew two and a half gallons of water from the legs of a small woman in three-fourths of an hour. I find it superior to all other modes. In the first place, it inflicts very little pain; secondly, it effectually relieves the cellular membrane of its aqueous ingorgement; thirdly, the wounds always heal kindly, producing very little or no inflammation, unlike that most cruel and *barbarous* old habit of whipping the legs with holly brushes, which produces a most distressing inflammation and troublesome sloughs and gangrene, like that of the lancet.

I prefer a No. 4 blunt new needle, that has never been used for any other purpose. I have it fixed in a piece of wood and hold it between the thumb and index finger, gauging the depth by the fingers, and then, by an active movement of the hand, puncture the skin, keeping out of the way of the larger veins and nerves and off of the shin bone, puncturing on either side and posteriorly to it, from the fact that I have observed, that any kind of a wound in the course of the anterior part of the tibia has a greater disposition to inflame than any other part of the leg.

